# Complete Genome Sequence and Methylome Analysis of *Bacillus globigii* ATCC 49760

**DOI:** 10.1128/genomeA.00427-16

**Published:** 2016-05-26

**Authors:** Richard D. Morgan

**Affiliations:** New England Biolabs, Ipswich, Massachusetts, USA

## Abstract

*Bacillus subtilis* (Ehrenburg) Cohn ATCC 49760, deposited as *Bacillus globigii*, is the source strain for the restriction enzymes BglI and BglII. Its complete sequence and full methylome were determined using single-molecule real-time (SMRT) sequencing.

## GENOME ANNOUNCEMENT

*Bacillus globigii* was originally provided by Gary A. Wilson, University of Rochester, NY, and is now housed in the ATCC (as ATCC 49760). It is the original source of the type II restriction enzymes (REases) BglI and BglII, first isolated in 1976 and sold commercially since 1978 (1–3). BglI recognizes and cleaves the bipartite DNA sequence GCCNNNN↓NGGC. BglII recognizes and cleaves the DNA sequence A↓GATCT. The complete genome sequence for *B. globigii* was determined using a Pacific Biosciences RSII instrument (PacBio, Menlo Park, CA) (4). One single-molecule real-time (SMRT) cell was used, generating 1,036 Mb of sequence, which was assembled into a single closed circular genome of 4,175,482 bp at an average coverage of 186-fold. Using the RS_Modification _and_Motif_Analysis.1 program of the SMRTPortal 2.3 analysis platform, two symmetrically methylated sequence motifs were detected: G^4m^**C**CNNNNNGGC, corresponding to the methylation target for M.BglI, and AGAT^4m^CT, corresponding to the methylation target for M.BglII, which are the methyltransferase components of the BglI and BglII restriction-modification (R-M) systems, respectively (**^4m^**C = N_4_-methylcytosine; underlined base indicates methylation [**^4m^**C] in the opposite strand). The SMRTPortal software called 98.8% of the BglI sites as modified and 97.7% of the BglII sites as modified, at an average coverage of 90× per motif (single strand of the DNA), indicating effectively complete modification for both R-M systems. The genes responsible for these modifications were identified and described previously by cloning the R-M systems (5, 6). In the automated GenBank annotation of the complete *B. globigii* genome sequence we determined, locus tag A1D11_20185 contains the M.BglI modification gene, and locus tag A1D11_20190 contains the BglI endonuclease gene of the BglI R-M system, while locus tag A1D11_20255 contains the M.BglII modification gene, locus tag A1D11_20260 contains the BglII endonuclease gene, and locus tag A1D11_20265 contains the C.BglII control gene of the BglII R-M system. The complete systems can be seen schematically on the REBASE database website (7). No additional DNA methyltransferase genes were found in the complete genome sequence by sequence similarity analysis in a comparison of putative *B. globigii* protein sequences to all known DNA methyltransferase protein sequences. A putative type IV methyl-directed restriction enzyme similar to 5mC-specific type IV R-M enzymes, such as EcoK Mrr or McrBC, is present in locus tag A1D11_00130.

### Nucleotide sequence accession number.

This complete genome sequence has been deposited in DDBJ/ENA/GenBank under the accession no. CP014840.
